# Association Between Job Characteristics, Benzene Exposure, and Hematopoietic Cancer Risk in Subway Maintenance Workers

**DOI:** 10.1016/j.shaw.2026.03.009

**Published:** 2026-04-04

**Authors:** Kanwoo Youn, Dong-Wook Lee, Ju-Hyun Park, Ro-Ting Lin, Dong-Uk Park

**Affiliations:** 1 Wonjin Institute for Occupational and Environmental Health, Republic of Korea; 2 Department of Preventive Medicine, Seoul National University College of Medicine, Seoul, Republic of Korea; 3 Institute of Environmental Medicine, Seoul National University Medical Research Center, Seoul, Republic of Korea; 4 Department of Statistics, Dongguk University, Jung-gu, Seoul, Republic of Korea; 5 Department of Occupational Safety and Health, College of Public Health, China Medical University, Taichung, Taiwan; 6 Department of Environmental Health, Korea National Open University, Republic of Korea

**Keywords:** Benzene, Hematopoietic cancer (HC), Job-exposure matrix (JEM), Subway maintenance workers (MWs), Thinners

## Abstract

**Background:**

The occurrence of multiple cases of hematopoietic cancers (HC) among subway train maintenance workers (MWs) at a municipally owned metro company prompted an investigation into their association with this maintenance work.

**Objectives:**

We aimed to evaluate the occurrence of HC among MWs relative to the general population and to examine the association between job characteristics, past benzene exposure, and HC risk.

**Methods:**

A cohort of 4,438 MWs was analyzed for the period 2005–2024. HC cases were identified through self-reporting and coworker reports obtained from a structured questionnaire survey. Diagnoses were confirmed via follow-up interviews. Respective benzene exposure was assessed using a job-exposure matrix (JEM) based on maintenance type and the historical use of thinners. Standardized incidence ratios were calculated to compare HC occurrence among MWs with that of the general population, and Cox proportional hazards ratio (HR) was employed to evaluate HC risk.

**Results:**

A total of eleven HC cases were identified, including leukemia (*n* = 4) and non-Hodgkin lymphoma (*n* = 7). The HC incidence risk among MWs was not significantly elevated compared with the general population. MWs classified with “definite” past benzene exposure probability had a significantly higher risk of HC than those with “possible” exposure (HR = 3.58, 95% confidence interval [CI] = 1.09–11.75**).** Although qualitative benzene exposure levels suggested a potential association with HCs (HR for “high” group = 3.09, 95% CI = 0.69–13.84 and HR for “moderate” = 2.22, 95% CI = 0.55-8.93 vs. “low”), the results were not statistically significant.

**Conclusion:**

Extended follow-up and additional case identification among MWs are required to clarify maintenance-specific associations with HC risk.

## 1 Introduction

Health issues among railroad workers, particularly cancer risks, have been investigated in occupational studies due to this group's exposure to diverse carcinogenic agents. Elevated lung cancer risks have been reported among railway maintenance personnel in the United States, likely associated with prolonged exposure to diesel engine exhaust emissions exposure during locomotive operation and repair tasks [1]. In addition, past usage of asbestos-containing materials in railcar maintenance has been linked to significantly increased incidence of mesothelioma and lung cancer in multiple nations [2]. An excess risk of hematopoietic cancer (HC) among train drivers in Switzerland has been suggested, related to chronic exposure to electromagnetic fields [3]. To our knowledge, while several epidemiological studies have investigated the association between benzene exposure and disease risks among maintenance workers in other industries [4,5], no studies have examined benzene exposure and HC risks among subway train maintenance workers.

A variety of chemical products, such as cleaning, painting, and coating agents, are routinely used in subway train maintenance. Some of these products are suspected to contain substances classified as Group 1 or Group 2A carcinogens by the International Agency for Research on Cancer (IARC), including benzene [6], trichloroethylene (TCE) [7], and dichloromethane (DCM) [8]. Numerous epidemiological studies have demonstrated that benzene exposure is strongly associated with an increased risk of HC, particularly leukemia [9] and non-Hodgkin lymphoma [10]. Benzene exposure and job characteristics in relation to HC risk among subway MWs estimated in our previous studies were applied to investigate the association between maintenance tasks, benzene exposure, and HC incidence [11,12]. The preceding studies concentrated on the characterization of maintenance-related exposures and the development of a retrospective, rule-based exposure assessment framework among subway MWs. In contrast, the present study builds upon this work by applying the exposure framework to a defined cohort to evaluate HC occurrence. This is achieved by using both external comparisons with the general population and internal risk analyses according to job characteristics and benzene exposure probability. The purpose of this study is to use previously developed a job-exposure matrix (JEM) and structured questionnaire data to retrospectively assess exposures to hematopoietic carcinogens, including benzene, among subway MWs [11,12], and to examine the associations between job-related exposures and the risk of HCs.

A number of cases of HC, including leukemia and non-Hodgkin lymphoma, have been reported among subway maintenance workers at Seoul Metro. It is noteworthy that a number of cases have been formally documented as occupational diseases, with a historical prevalence of benzene-containing cleaning agents in workshops with inadequate ventilation being identified [13]. Subway MW involves tasks with potential chemical exposures from cleaning, painting, and lubrication, and an investigation was initiated to evaluate the occurrence of HC and its association with maintenance tasks. In response to these concerns, Seoul Metro initiated an investigation into the prevalence of HC among subway MWs and any potential association with their maintenance tasks.

## 2 Methods

### 2.1 Overview of maintenance procedures for subway trains

Our previous study provided an overview of the subway train maintenance process , which forms the basis for the retrospective exposure assessment presented here. Subway train maintenance includes light maintenance (routine inspections) and heavy maintenance (full overhauls every 3–4 years). Heavy maintenance has involved extensive cleaning using degreasers and petroleum-based solvents, including benzene-containing thinners and diesel oil, which were commonly used until 2011. In that year, safer alternatives such as SUPER-3000 and water-based products were introduced under chemical substitution policies. Oil-based paints and their associated thinners were further phased out by 2022 due to health concerns. The relatively stable job roles and limited worker mobility among the subjects support the reliability of exposure classification by maintenance type.

### 2.2 Description of study subjects and hematopoietic cancer (HC) case identification

The study population included a total of 4,438 subway train MWs employed between 2005 and 2024 (Table 1). A cohort backbone was constructed using company personnel records encompassing all workers who had been employed during this period. The date of entry was defined as the earliest date from 2005 onward at which employment could be confirmed from personnel records, while the end date was defined as the date of HC diagnosis, death, or December 31, 2024, whichever occurred first.

**Table 1:** Characteristics of subway train maintenance workers (MWs) included in the study (2005–2024, *N* = 4,438; 62,482 person-years) extracted from our previous study [12] and presented by current and retired status for cohort description and case ascertainment purposes

Classification	Current MWs	Retired MWs	Total
**Structured questionnaire survey**			
Respondents	2,801	114	2,915
Non-respondents∗	296	1,227	1,523
**Age group (years)**			
≤30	270	0	270
31-40	496	0	496
41-50	342	2	344
51-60	1,660	40	1,700
>60	32	75	107
Non-respondents∗	297	1,224	1,521
**Year of first employment**			
1982-1990	82	677	759
1991-2000	1,677	524	2,201
2001-2010	236	49	285
2011-2020	796	78	874
>2020	306	13	319
**Total**	3,097	1,341	4,438

∗ Due to non-responses to the questionnaire, detailed information on age and MW job changes during employment may be unavailable. Sex was not included as a variable, as the study population was predominantly male.

As systematic registry linkage for all retired workers was not a viable option, HC cases were identified using a range of complementary sources, including occupational disease compensation claim records, self-reported diagnoses from structured questionnaires, coworker reports of retired workers, and reviews of medical leave and medical examination records. A separate follow-up survey targeting retired workers was conducted through the labor union. Additionally, information about past HC diagnoses among former employees was gathered indirectly from current employees who reported knowing coworkers previously diagnosed with HC. This multi-source strategy was developed to maximize case ascertainment and minimize under-ascertainment in a cohort of small companies. No cases of HC were diagnosed within the first year of employment (<1 year): exclusion of these potential short-latency cases did not materially change the risk estimates. For all reported HC cases obtained through the questionnaire or indirect coworker testimonies, diagnoses were further verified by physicians who directly confirmed the diagnosis details and relevant medical history via telephone interviews.

### 2.3 Cohort period definition and temporal analysis

The cohort included subway maintenance workers employed during any period between 2005 and 2024. To examine temporal patterns, employment duration was categorized into tertiles: ≤18 years, 19–30 years, and >30 years. No lag time was applied, primarily because the number of HC cases was small (*n* = 11), and HC may develop within relatively short latency periods (e.g., <5 years) following benzene exposure, making long lag assumptions less appropriate. Exposure assessment covered each worker's entire employment history, thereby reflecting both cumulative and time-varying exposures. Similar approaches without lag periods have been reported in previous benzene–HC epidemiological studies with comparable methodological and latency considerations [9,14].

### 2.4 Retrospective assessment of benzene exposure among MWS: job profiles, exposure prevalence, and levels

In our two previous studies, we provided detailed descriptions of subway train maintenance processes, identified hematopoietic carcinogens, including benzene [11], and developed retrospective assessment methods for benzene exposure and maintenance work characteristics among MW [12]. We briefly summarize them again here to examine the association between maintenance work and HC risk among MWs (Table 2 and Table 3). The retrospective benzene exposure assessment was based on three primary information sources, namely: (1) structured questionnaires administered to current and retired maintenance workers, (2) focus group interviews with maintenance team leaders and labor union representatives, and (3) company personnel records. The questionnaire collected detailed information on job history, maintenance tasks, employment periods, and self-reported health outcomes, including HC diagnoses. To facilitate self-administration, the final questionnaire was implemented using a web-based platform built upon the R-Shiny framework [15], with responses automatically recorded to a linked Google Sheet [16]. Both distribution and response collection were conducted online, while the purpose and instructions of the survey were explained in person prior to completion. Information on past occupational exposure and major health outcomes, including HC, was collected through this structured questionnaire administered to current and former subway train MWs. The related labor union assisted in encouraging retired workers to participate.

**Table 2:** Key chemicals and maintenance work details for retrospective benzene exposure assessment in subway maintenance workers (MWs)

Employment period	A brief description of the use of chemical products that may contain benzene.	Corresponding MW	Exposure probability level∗	Estimated qualitative exposure level to benzene [17] ∗
Prior to 2011	Thinners and diesel were estimated to be widely used for cleaning train components, work floors, etc. Thinners were also used to dilute oil-based paint.	All heavy MW	Definite	High
2011-2022	Since 2011, thinners and diesel have been replaced as cleaning agents by SUPER 3000, which does not contain benzene. However, thinners were widely used to dilute paint.	Exclusive heavy MWs who performed painting	Definite	High
		Other heavy MWs, except painters.	Probable	Moderate
Since 2023	Since 2023, oil-based paints that require thinners have been replaced with water-based paints. Spray chemical products that may contain polluted levels of benzene are estimated to clean and lubricate parts irregularly in heavy and light MW.	All heavy MW	Possible	Low
Past to present	Spray chemical products that may contain benzene have been widely used to clean and lubricate parts irregularly in heavy and light MW. The level of benzene in spray products varies over time.	All MWs using spray-type chemical products for cleaning and lubrication.	Possible	Moderate or low

∗ The three-level classification presented as an example is arbitrary and subjective, as it could vary depending on factors such as the evaluator's judgement, the properties of the chemical product containing benzene, and exposure conditions during maintenance work.

**Table 3:** Combined information on maintenance work and chemical use for estimating past benzene exposure among subway maintenance workers (integrated tables from Park's study)

Exposure category	Exposure subcategory∗	Brief description for retrospective exposure assessment
Chemical products	Paint thinners & cleaners	Benzene-containing thinners used until 2011 (cleaning and painting); only for painting until 2022; not used from 2023 onward
	Oil-based paints	Gradually substituted by water-based paints around 2023
	Spray products	Continuous use of spray products and other chemicals for lubrication and cleaning from past to present
		Spray chemical products that may contain benzene have been widely used to clean and lubricate parts irregularly in heavy and light MW. The level of benzene in spray products varies over time.
Air monitoring results	Benzene levels	All airborne measurements far below OEL (0.05 ppm)
		Since 2023, oil-based paints that require thinners have been replaced with water-based paints. Spray chemical products that may contain polluted levels of benzene are estimated to clean and lubricate parts irregularly in heavy and light MW.
Job characteristics	Maintenance type	Heavy maintenance work (MW): Involves overhauls and extensive cleaning using chemical products, including thinners and sprays, as well as diluting oil-based paints with thinners.
		Light maintenance work (MW): Consists of routine inspections and minor repairs, often involving the use of spray chemical products.
		Office work: Refers to administrative duties without direct chemical exposure.
	Year of first employment	Before 2011, thinners containing benzene were widely used for both cleaning and paint dilution.
		From 2011 to 2022, their use was limited primarily to oil-based paint dilution.
		After 2022, benzene-free products were introduced, except for a few spray products that were found to contain trace benzene contamination.
	Work duration	≥5 years vs. <5 years
Benzene exposure classification based on JEM incorporating maintenance type, work duration, and year of first employment	Probability level	Definite: exclusive heavy MW before 2011, Possible: All job types except exclusive heavy maintenance work prior to 2011
	Intensity level	High: workers who had more than five years of exclusive maintenance work experience and were employed prior to 2011
		Moderate: workers who performed both heavy and light maintenance work for over five years and were employed prior to 2012.
		Low: Either workers either exclusively engaged in light maintenance and office work since employment, or workers employed prior to 2011 with <5 years of combined heavy and light maintenance work experience

∗ ∗The classification presented is arbitrary and subjective, as it could vary depending on factors such as the evaluator's judgement, the properties of the chemical product containing benzene, and exposure conditions during maintenance work.

Key exposure-related variables included maintenance work type (heavy, light, or mixed), changes in work type over time, year of first employment, employment duration by maintenance type, and periods of benzene-containing thinner use. The substance was initially employed for cleaning and painting purposes until 2011, subsequently for painting only until 2022, and was to be discontinued after 2023, with the exception of trace contamination in certain spray products. -

Using these variables, a time-stratified, semi-quantitative JEM was developed, based on explicit decision rules, to evaluate maintenance work characteristics and to assign the probability and intensity of past benzene exposure. The JEM integrated maintenance job type, duration of employment, and historical changes in the use of benzene-containing products are used to systematically classify exposure across predefined job and time strata. Workers exclusively engaged in heavy maintenance before 2011 were classified as having a “definite” exposure probability, whereas all others were classified as “possible.” The workers classified as having a “possible” probability of past benzene exposure constituted a heterogeneous, conservatively defined reference group. This group included individuals with intermittent, low-level, or no direct exposure. It was used to represent lower and uncertain exposure probability in order to avoid misclassification in the absence of detailed historical measurement data. Although questionnaire respondents provided detailed job histories, information for non-respondents was limited to job titles at the time of hiring and categorized as “unclassifiable.” Workers for whom inadequate information was available for the purposes of classification were retained in the analytic dataset as “unclassifiable,” rather than excluded, in order to avoid selection bias due to differential availability of job histories (particularly among non-respondents and retired workers). This approach aimed to maximize the inclusion of HC cases and exposed maintenance workers in the analysis.

Subway MWs were semi-qualitatively classified according to the JEM into two probability groups of past benzene exposure (“definite” and “possible”) and three semi-qualitative exposure level groups (“high,” “medium”, and “low”) (Table 2). First, employment periods were divided into before and after 2011. Second, workers were grouped by employment duration (≥5 years vs. <5 years). Finally, job types were classified as heavy maintenance only, mixed heavy and light maintenance, or light maintenance only. Benzene exposure levels for each group were assessed as three categories (high, medium, and low). Since 2023, when oil-based paints were replaced with water-based alternatives, benzene exposure has been assessed as “low,” as only a few benzene-contaminated spray products were found to be used infrequently. The classification and evaluation of past exposures to chemicals and maintenance work characteristics potentially associated with HCs, including benzene, were performed independently and without prior knowledge of the participants' cancer status. This approach ensured that the retrospective exposure assessment was not biased by the known occurrence of HC cases among MWs.

### 2.5 Assessment of the association between subway train maintenance work and HC risk

The risk of HC among subway train MWs was evaluated using two analytical approaches. First, the incidence of HC in the MW cohort was compared with that of the general population using the indirect standardization method, adjusting for age distribution. Specifically, standardized incidence ratios (SIRs) were calculated using indirect standardization, considering five-year age intervals to address differences in age distribution between groups. Person-years for each age category were computed based on the age at cohort entry and at the end of follow-up. The standardized population selected was the 2014 male population, with cancer incidence rates by five-year age groups obtained from annual cancer registration data published by the Korean Ministry of Health and Welfare. To examine potential variations arising from the choice of standard population, sensitivity analyses were conducted using alternate reference populations from 2005 to 2022, the most recent year for which national cancer incidence data were available. SIRs were derived for HCs (ICD-10 codes C81–C96, including Hodgkin lymphoma, non-Hodgkin lymphoma, multiple myeloma, and leukemia), utilizing age-specific incidence rates from national statistics. Ninety-five percent confidence intervals (95% CIs) for the SIRs were calculated assuming a Poisson distribution of cancer events, as described by Rothman and Greenland [18]. Second, an internal comparison was conducted within the MWs population by analyzing HC occurrence in relation to retrospective exposure variables, such as job tasks and estimated levels of benzene exposure. Cox proportional hazards models were applied to estimate hazard ratios (HRs) and 95% confidence intervals (CIs) for HC across the duration of employment in maintenance roles. Person-time was calculated from the start of employment or 2005 (whichever was later) to the date of HC diagnosis, death, or the end of follow-up (2024), whichever came first. In the Cox proportional hazards modeling, age was utilized as the underlying time scale. All models were adjusted for age group. Due to the limited number of HC cases, the primary application of the Cox proportional hazards models was to explore the direction and magnitude of associations between retrospective exposure variables and HC risk, rather than to derive definitive causal estimates. The age variable was utilized as the fundamental time scale, and the majority of exposure variables were assessed employing univariate models to avoid model instability resulting from sparse data. Given the very small number of HC cases, all exposures, including benzene, were modeled as fixed baseline covariates rather than time-dependent variables. The proportional hazards assumption was evaluated using Schoenfeld residual-based tests and graphical inspection; no major violations were detected, although statistical power was limited due to the small number of cases. All statistical analyses were performed using the latest version of R 4.3.2. (R Core Team, 2025). The study protocol was approved by the Institutional Review Board (IRB No. 1253-202501-HR-001-01) to ensure compliance with ethical standards, including the protection of personal information and informed consent procedures.

## 3 Results

**Fig. 1:**
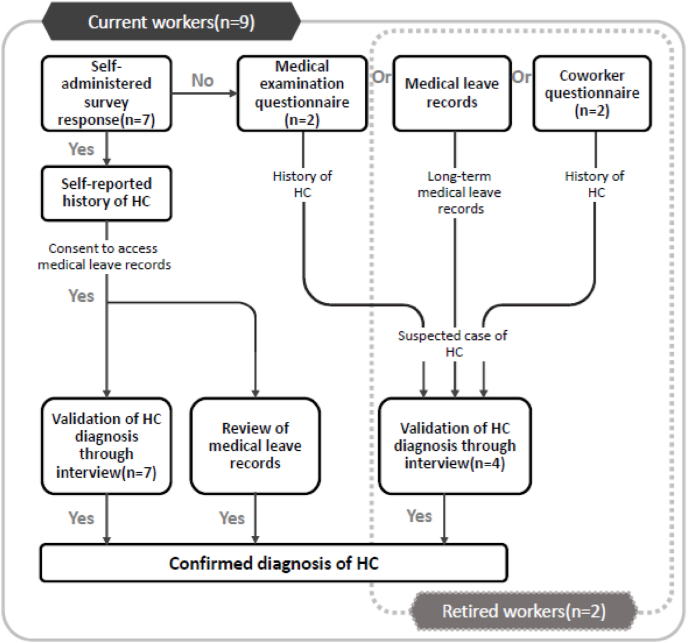
Schematic diagram of the process used to identify hematopoietic cancer (HC) cases among subway maintenance workers, including data collection through questionnaires, medical sick leave records, and medical examination results, with diagnosis. Retired workers are shown to illustrate additional case identification pathways.

The total follow-up period accumulated across all participants was 62,482 person-years. In total, 2,915 workers (66%) responded to the structured questionnaire (Table 1). A total of eleven HC cases were identified among the 4,438 MWs. Four cases had previously been reported through industrial accident compensation claims, while seven new cases were identified—five via self-reporting and two through coworker reports of retired colleagues obtained from this study's questionnaire-with overlap across sources resolved by counting each confirmed case once after physician verification. Retired workers were not analyzed as a separate group, but rather included solely to enhance the identification of HC cases that occurred during employment. (Table 4 and Fig. 1). Among the eleven HC cases, seven current MWs provided detailed job histories, and all HC cases were found to have developed during employment. The overall risk of HC among the MWs, including both heavy and light MWs groups, was not significantly higher than that of the general population (Table 5). Separate analyses of lymphoma and leukemia also showed no statistically significant risks (data not shown). However, Cox proportional hazards analysis within the MW cohort indicated that MWs classified with a “definite” probability of past benzene exposure had a significantly higher risk of HC compared with those classified as “possible” (HR = 3.58, 95% CI = 1.09–11.75), suggesting a potential association between benzene exposure and HC development. No significant associations were observed for other JEM variables, such as estimated benzene exposure levels, type of maintenance work (e.g., heavy vs. light), or year of first employment. Although qualitative benzene exposure levels suggested a potential association with HCs (HR for “high” group = 3.09, 95% CI = 0.69–13.84; HR for “moderate” = 2.22, 95% CI = 0.55-8.93 vs “low”), the results were was not statistically significant (Table 6), likely due to the small number of HC cases (*n* = 11) and the limited cohort size. Subgroup-specific HR estimates, including those for age categories, were based on a very small number of cases and should therefore be interpreted with caution.

**Table 4:** Job history, cancer type, and diagnosis year of hematopoietic cancer (HC) cases among subway maintenance workers (MWs)

Employed status	The year of diagnosis for HC	The year of first employment	Latency period (years) ∗	Type of HC	Type of MW involved in during employment	Methods of identifying HC cases among MWs	Structured survey response
Current	2007	1984	23	Acute leukemia	Exclusive heavy MW	Occupational disease compensation claim records	No
Current	2012	1996	16	Non-Hodgkin lymphoma	Exclusive heavy MW	Occupational disease compensation claim records	Yes
Current	2015	1998	17	Non-Hodgkin lymphoma	Exclusive heavy MW	Occupational disease compensation claim records	No
Current	2019	1997	22	Non-Hodgkin lymphoma	Exclusive light MW	Self-administered survey response	Yes
Current	2022	1992	30	Non-Hodgkin lymphoma	Exclusive heavy MW	Self-administered survey response	Yes
Current	2023	1997	26	Non-Hodgkin lymphoma	Both	Occupational disease compensation claim records	Yes
Current	2007	1990	17	Chronic leukemia	Exclusive light MW	Self-administered survey response	Yes
Current	2021	1995	26	Malt lymphoma	Both	Self-administered survey response	Yes
Current	2009	2002	7	Non-Hodgkin lymphoma	Exclusive light MW	Self-administered survey response	Yes
Retired	2009	1993	16	Acute leukemia	Exclusive heavy MW	Coworker questionnaire	No
Retired	2009	1995	18	Acute leukemia	Exclusive light MW	Coworker questionnaire	No

Abbreviation: HC, hematopoietic cancer; MW, maintenance work.

∗ All HC cases were diagnosed during employment, and latency periods were calculated from the year of first employment to the year of diagnosis.

**Table 5:** Standardized incidence ratio (SIR) for hematopoietic cancers∗ among subway train maintenance workers by reference population year

Reference population year	SIR	95% confidence interval
2005	1.28	(0.64 – 2.30)
2006	1.28	(0.64 – 2.29)
2007	1.26	(0.63 – 2.25)
2008	1.26	(0.63 – 2.25)
2009	1.24	(0.62 – 2.22)
2010	1.24	(0.62 – 2.22)
2011	1.22	(0.61 – 2.18)
2012	1.2	(0.60 – 2.14)
2013	1.13	(0.57 – 2.03)
2014	1.13	(0.57 – 2.03)
2015	1.1	(0.55 – 1.97)
2016	1.09	(0.55 – 1.96)
2017	1.14	(0.57 – 2.05)
2018	1.05	(0.53 – 1.89)
2019	1.02	(0.51 – 1.83)
2020	1.09	(0.55 – 1.95)
2021	1.04	(0.52 – 1.85)
2022	0.97	(0.49 – 1.74)
2005∼2022 cumulated	1.15	(0.57 – 2.05)

∗ Hematopoietic cancers include leukemia (*n* = 4) and non-Hodgkin's lymphoma (*n* = 7) (Table 4).

**Table 6:** The association between hematopoietic cancer (HC) risk and maintenance work (MW)-related exposure variables, with a focus on benzene, using Cox proportional hazards model

Exposure variable	Exposure category	*n*	%	Total person-years of follow-up	No. of HCs	Hazard ratio†	95% confidence interval
Age group	≤30	955	21.5	6719	0	N/A§	
	31-40	2317	52.2	39,419	9	1.34	(0.27 - 6.73)
	41-50	1011	22.8	15,493	2	1	(Reference)
	>50	155	3.5	923	0	N/A	
Employment duration	≤ 18	1519	34.2	10,341	2	1	(Reference)
	19-30	1598	36.0	27,795	7	0.58	(0.11 - 3.05)
	>30	1319	29.7	24,378	2	0.20	(0.02 - 1.56)
	No classification	2	0.1	40	0	N/A	
Year of first employment	<2011	3271	73.7	55,954	11	1	(Reference)
	Since 2011	1167	26.3	6601	0	N/A	
Type of job performed since initial employment year	Exclusive heavy MW since initial employment <2011	305	6.9	5589	1	1	(Reference)
	Both MW since initial employment <2011	1636	36.9	31,818	6	1.08	(0.13 - 8.94)
	Exclusive heavy MW employed since 2011	425	9.6	2288	0	N/A	
	Both MWs employed since 2011	549	12.4	3412	0	N/A	
	No classification	1523	34.3	19,448	4	1.91	(0.19 - 18.7)
Employment duration by MW type	Exclusive heavy MW (>10 years)	619	14.0	11,213	5	1	(Reference)
	Mixed light and heavy MW (5–10 years)	280	6.3	3179	0	N/A	
	Mixed light and heavy MW (<5 years)	2020	45.5	28,737	6	0.51	(0.16 - 1.68)
	No classification	1519‡	34.2	19,426	0	N/A	
Type of MW	Exclusive heavy MW	788	17.8	8490	3	1	(Reference)
	Both	975	22.0	15,270	4	0.68	(0.15 - 3.03)
	No MW	2122	47.8	31,426	4	0.34	(0.08 - 1.51)
	No classification	553	12.5	7368	0	N/A	
Ever performed heavy MW	Yes	1477	33.3	20,196	7	2.01	(0.59 - 6.87)
	No	1442	32.5	22,932	4	1.00	(Reference)
	No classification	1519‡	34.2	19,426	0	N/A	
Probability level of past benzene exposure.	Definite: Involved exclusively in heavy MW prior to 2011∗	537	12.1	10,093	6	3.58	(1.09 – 11.75)
	Possible: All job types except exclusive heavy maintenance work prior to 2011	2382	53.7	33,035	5	1	(Reference)
	No classification	1519‡	34.2	19,426	0	N/A	
Estimated past benzene exposure level	High	296	6.7	5389	3	3.09	(0.69 - 13.84)
	Moderate	562	12.7	10,948	4	2.22	(0.55 - 8.93)
	Low	2061	46.4	26,791	4	1	(Reference)
	No classification	1519‡	34.2	19,426	0	N/A	

∗ Prior to 2012, when thinners containing benzene were still used for cleaning and as paint diluents, benzene exposure prevalence was estimated to be definite.

† Hazard ratios (HRs) and 95% confidence intervals (CIs) for each univariate exposure variable were adjusted for age group.

‡ Among the 1,523 non-respondents, 4 cases were included due to sufficient job data, but 1,519 remained unclassified.

§ NA; not applicable.

## 4 Discussion

This study demonstrated that “definite” past benzene exposure—defined by exclusive engagement in heavy maintenance work prior to 2011—was significantly associated with an elevated risk of HC. This exposure category is likely to reflect a broader historical high-hazard maintenance environment rather than benzene exposure alone, as heavy maintenance during this period involved concurrent exposure to multiple solvents and process-related hazards, including benzene-containing thinners, diesel, and other organic solvents. However, in the retrospective cohort of subway maintenance workers (*n* = 4,438), no significant associations were observed between other JEM variables—including qualitative benzene exposure estimates—and maintenance work type, employment period, year of initial employment, and exposure level (Table 6). Our findings are consistent with the existing literature on benzene exposure and HC risk, although the estimates should be interpreted as conservative due to the limited number of cases and potential non-differential exposure misclassification. Previous occupational cohort studies and meta-analyses have consistently reported increased risks of leukemia and certain lymphoma subtypes associated with benzene exposure across diverse industries [9,19]. These findings highlight the need for extended follow-up and additional HC case identification, particularly among retired workers, to more precisely evaluate HC risk in relation to detailed maintenance job profiles. Nevertheless, this study provides an important foundation for future epidemiological research focusing on maintenance job-specific exposures and HC risks within a relatively small population drawn from a single company. Key challenges encountered in developing JEMs for hematopoietic carcinogen exposure and applying them to the assessment of HC risk are discussed here.

First, our key finding that workers with a higher probability of benzene exposure had a significantly elevated HR underscores benzene's role as a major hematopoietic carcinogen contributing to HC risk among subway train MWs. However, no significant associations were observed between benzene exposure levels and HC risk, likely due to the small number of HC cases (*n* = 11) (Table 4), the relatively small size of the MW cohort, and the limited response rate to our structured questionnaire. Unlike with large-scale population-based or industry-wide HC epidemiological studies [20], a relatively small cohort of MWs from a single facility or company poses challenges in establishing both association and causal relationships due to the limited number of cases and difficulties in tracking HC cases among retired workers (Table 1, Table 4). In our analysis, statistically significant variables were difficult to identify because the HR estimates for various job categories and benzene exposure levels had wide 95% CIs. For example, the HRs for semi-qualitative benzene exposure levels were meaningfully elevated—2.75 (95% CI: 0.7–13.8) for the high-exposure group and 2.22 (95% CI: 0.55–8.93) for the moderate-exposure group—compared to the reference group, although these results were not statistically significant (Table 6). Nevertheless, the elevated HRs suggest a potential association with increased HC risk among MWs with higher benzene exposure, which is consistent with previous occupational studies that reported similar patterns despite wide CIs in small cohorts [21]. These findings underscore the potential utility of small-scale cohort studies in identifying job-related cancer risks within specific companies or occupational settings.

Secondly, in the absence of detailed job histories and exposure records, the time-stratified JEM — based on documented changes in solvent use, notably the 2011 substitution of benzene-containing thinners—proved essential for reconstructing historical exposures and provides a practical methodological framework for similar occupational epidemiology studies [17,22]. In this study, JEMs were developed using MWs' questionnaire responses, company documentation on chemical purchases, and group interviews. Given the scarcity and limited reliability of company-held measurements (Table 2, Table 3), these JEMs integrated data on maintenance work type, year of initial employment, employment duration, and the period of benzene-containing chemical use to estimate both qualitative benzene exposure levels and probability. For example, workers in heavy maintenance for more than five years before 2011 were estimated to have a ‘high’ cumulative qualitative benzene exposure. This approach provides an alternative method for investigating the long-term risk of HC from benzene in the absence of a representative cumulative exposure level. When comprehensive records of work history, benzene measurements, or chemical usage were unavailable, we relied not only on the structured questionnaires completed by all MWs, but also on focus focus group interviews with MWs to obtain detailed information on job tasks and health outcomes including HC.

Thirdly, multiple case-finding strategies, including structured questionnaires, coworker reports, medical examination records, and sick-leave data, were used to maximize case ascertainment and minimize under-ascertainment bias (Fig. 1 and Table 4). Four HC cases who did not participate in the questionnaire survey were proactively identified through coworker reports and medical leave records, in addition to information from self-administered questionnaires. Moreover, the large cohort and long follow-up period (62,482 person-years from 2005 to 2024) enabled robust evaluation of HC incidence over time. This multi-source case ascertainment approach reduces potential under-reporting compared to studies relying solely on questionnaires and can be effectively applied in small-company or community-based epidemiological research. Nevertheless, under-ascertainment of cases, particularly among retired workers, may have occurred, which would be expected to bias risk estimates towards the null rather than exaggerate observed associations.

Finally, in this study, leukemia and lymphoma cases were combined and analyzed collectively as HCs due to the small number of individual cases, which limited statistical power. This approach is epidemiologically justifiable, as both leukemia and certain types of lymphoma share common occupational risk factors, particularly exposure to benzene and related hematopoietic carcinogens [6]. Previous occupational cohort studies have also grouped hematopoietic malignancies when evaluating solvent exposure risks, demonstrating similar associations [14,21]. In situations where the number of individual leukemia or lymphoma cases is limited—such as in specific companies or industrial processes—it is methodologically sound to combine hematopoietic malignancies into a single outcome category, as done in this study, to improve statistical power. This approach is supported by previous occupational epidemiology research demonstrating that combining leukemia with lymphoid neoplasms facilitates more robust risk estimation when sample sizes are small. Large cohort studies in petroleum and offshore industries have reported that occupational benzene exposure increases the mortality risk of lymphohematopoietic malignancies [20], which partially supports our approach of combining HC types. However, differences in pathogenesis and latency between leukemia and lymphoma warrant cautious interpretation, and future studies with larger case numbers should analyze these outcomes separately.

A major limitation of this study is the small number of HC cases (*n* = 11), which restricts statistical power and precision of HR estimates, particularly in subgroup analyses. Therefore, the Cox proportional hazards results should be interpreted as exploratory. Furthermore, although exposure conditions changed over time and benzene exposure was modelled as a fixed baseline covariate, the limited number of cases prevented a formal assessment of the proportional hazard assumption. Any unmodelled time-varying effects are likely to have resulted in conservative rather than inflated HR estimates. Furthermore, incomplete retrospective exposure data—particularly for the approximately 1,523 non-respondents—precluded the construction of a detailed JEM and may have introduced exposure misclassification. Exposure misclassification among non-respondents classified as “unclassifiable” is likely non-differential due to exposure assessment and would therefore attenuate HRs toward the null, indicating that the observed associations—particularly for the “definite” exposure probability group—are conservative. Nevertheless, the study has notable strengths, including the proactive identification of HC cases within a relatively small cohort from a single workplace and the demonstration of a potential association between benzene exposure and HC risk using a subway maintenance–focused JEM that incorporates documented changes in solvent use over time and maintenance-specific job characteristics.

In conclusion, this study identifies a significant association between definite past benzene exposure and HC risk among MWs. The elevated risk observed in the definite exposure group is biologically plausible and consistent with findings from previous occupational studies of benzene-exposed workers. Further follow-up is required to identify additional cases of HC, in conjunction with a more detailed retrospective exposure assessment. This will facilitate a more robust evaluation of job-specific risks and enhance causal inference.

## CRediT authorship contribution statement

**Kanwoo Youn:** Validation, Methodology, Formal analysis, Data curation. **Dong-Wook Lee:** Validation, Resources, Methodology, Investigation, Formal analysis, Data curation. **Ju-Hyun Park:** Writing – original draft, Validation, Methodology, Investigation, Formal analysis, Data curation. **Ro-Ting Lin:** Validation, Resources, Methodology. **Dong-Uk Park:** Writing – review & editing, Writing – original draft, Visualization, Validation, Supervision, Software, Resources, Project administration, Methodology, Investigation, Funding acquisition, Formal analysis, Data curation, Conceptualization.

## Statement on the use of AI tools

Generative AI tools were used to rephrase sections of the manuscript for improved clarity during its preparation. However, the authors developed and validated all scientific content, interpretation of results and conclusions. The authors take full responsibility for the content and integrity of the work.

## Conflicts of interest

The authors whose names are listed immediately below certify that they have NO affiliations with or involvement in any organization or entity with any financial interest (such as honoraria; educational grants; participation in speakers' bureaus; membership, employment, consultancies, stock ownership, or other equity interest; and expert testimony or patent-licensing arrangements), or non-financial interest (such as personal or professional relationships, affiliations, knowledge or beliefs) in the subject matter or materials discussed in this manuscript.
